# Sensitivity and Specificity of Dual-Isotope ^99m^Tc-Tetrofosmin and ^123^I Sodium Iodide Single Photon Emission Computed Tomography (SPECT) in Hyperparathyroidism

**DOI:** 10.1371/journal.pone.0129194

**Published:** 2015-06-16

**Authors:** Michael Sommerauer, Carmen Graf, Niklaus Schäfer, Gerhard Huber, Paul Schneider, Rudolf Wüthrich, Christoph Schmid, Hans Steinert

**Affiliations:** 1 Department of Nuclear Medicine, University Hospital Zurich, Ramistrasse 100, 8091, Zurich, Switzerland; 2 Department of Otorhinolaryngology, Head and Neck Surgery, University Hospital Zurich, Frauenklinikstrasse 24, 8091, Zurich, Switzerland; 3 Department of General, Visceral and Transplant Surgery, University Hospital Zurich, Ramistrasse 100, 8091, Zurich, Switzerland; 4 Division of Nephrology, University Hospital Zurich, Ramistrasse 100, 8091, Zurich, Switzerland; 5 Division of Endocrinology and Diabetology, University Hospital Zurich, Ramistrasse 100, 8091, Zurich, Switzerland; Uppsala University, SWEDEN

## Abstract

**Purpose:**

Despite recommendations for ^99m^Tc-tetrofosmin dual tracer imaging for hyperparathyroidism in current guidelines, no report was published on dual-isotope ^99m^Tc-tetrofosmin and ^123^I sodium iodide single-photon-emission-computed-tomography (SPECT). We evaluated diagnostic accuracy and the impact of preoperative SPECT on the surgical procedures and disease outcomes.

**Methods:**

Analysis of 70 consecutive patients with primary hyperparathyroidism and 20 consecutive patients with tertiary hyperparathyroidism. Imaging findings were correlated with surgical results. Concomitant thyroid disease, pre- and postoperative laboratory measurements, histopathological results, type and duration of surgery were assessed.

**Results:**

In primary hyperparathyroidism, SPECT had a sensitivity of 80% and a positive predictive value of 93% in patient-based analysis. Specificity was 99% in lesion-based analysis. Patients with positive SPECT elicit higher levels of parathyroid hormone and higher weight of resected parathyroids than SPECT-negative patients. Duration of parathyroid surgery was on average, approximately 40 minutes shorter in SPECT-positive than in SPECT-negative patients (89±46 vs. 129±41 minutes, p=0.006); 86% of SPECT-positive and 50% of SPECT-negative patients had minimal invasive surgery (p = 0.021). SPECT had lower sensitivity (60%) in patients with tertiary hyperparathyroidism; however, 90% of these patients had multiple lesions and all of these patients had bilateral lesions.

**Conclusion:**

Dual-isotope SPECT with ^99m^Tc-tetrofosmin and ^123^I sodium iodide has a high diagnostic value in patients with primary hyperparathyroidism and allows for saving of operation time. Higher levels of parathyroid hormone and higher glandular weight facilitated detection of parathyroid lesion. Diagnostic accuracy of preoperative imaging was lower in patients with tertiary hyperparathyroidism.

## Introduction

In patients with hyperparathyroidism (HPT), accurate preoperative localization of the hyperactive parathyroid lesions is essential for planning minimal invasive surgery. Parathyroid imaging has been shown to be an effective tool in the preoperative localization of the dominant source of the parathyroid hormone (PTH) excess [1, 2]. Several techniques have been introduced, the most common are the dual-phase imaging using ^99m^Tc-sestamibi and the dual-tracer imaging approach using ^99m^Tc-sestamibi in combination with ^99m^Tc-pertechnetate or ^123^I sodium iodide; however, only the latter combination can be recorded simultaneously [3, 4]. In direct comparison of both methods, the dual-tracer method elicited a 5–10% higher sensitivity [3, 5–7]. The dual-tracer technique enables a ^99m^Tc-tetrofosmin / ^123^I sodium iodide subtraction single-photon-emission-tomography (SPECT) for more precise localization of parathyroid lesions. However, in a more recent study, dual-tracer SPECT imaging using ^99m^Tc-sestamibi in combination with ^123^I sodium iodide showed a relatively low sensitivity of 71% and a disappointing specificity of 48% [8].

^99m^Tc-tetrofosmin has similar uptake kinetics as ^99m^Tc-sestamibi in the thyroid and parathyroid glands but only ^99m^Tc-sestamibi has different washout kinetics in thyroid and parathyroid tissue [9]. Hence, in current guidelines, the use of ^99m^Tc-tetrofosmin is recommended for the dual-tracer imaging only [4]. Surprisingly, no study on dual-isotope imaging of ^99m^Tc-tetrofosmin in combination with ^123^I sodium iodide in HPT is available so far.

Therefore, we analyzed the accuracy of dual-isotope ^99m^Tc-tetrofosmin and ^123^I sodium iodide SPECT in the detection of parathyroid lesion in HPT, and compared the imaging results with the clinical and surgical findings

## Methods

### Patients

We screened all consecutive patients undergoing dual-isotope ^99m^Tc-tetrofosmin and ^123^I sodium iodide SPECT between 01/2005 and 12/2013 (n = 255). One-hundred ten patients had SPECT prior to parathyroid surgery at the University Hospital Zurich and were eligible for further analysis. From this total, we excluded 20 patients due to insufficient data (no pre- and post-operative PTH levels documented). We additionally assessed clinical, laboratory and surgical data. Diagnosis of primary (p-HPT, consistently with hypercalcemia) versus tertiary hyperparathyroidism (t-HPT, most commonly with a history of renal disease) was done according to current clinical guidelines by specialists in endocrinology and nephrology. Demographic information, information on concomitant thyroid disease (as determined by thyroid sonography or elevated thyroid antibodies) and laboratory values (pre-operative serum values of parathyroid hormone (PTH), calcium, phosphate, and creatinine) were collected. PTH levels higher than 70 ng/l were considered as pathological [10]. We included post-operative laboratory values within an interval of up to 3 months after surgery. Surgery was performed by experienced endocrine surgeons after discussion of SPECT imaging results at the interdisciplinary thyroid board at our institution. Type of operation (minimal invasive versus bilateral neck exploration) was chosen by discretion of the endocrine surgeon; total duration of surgery was assessed. In 3 patients, operation duration was not recorded; 7 patients received combined parathyroid and thyroid intervention, and operation duration was therefore not taken into consideration. If SPECT was negative, further imaging was allowed by discretion of the endocrine surgeon to facilitate minimal invasive surgery (including sonography, computed tomography (CT) and magnetic resonance imaging. Histopathological work-up of surgery specimens and assessment of weight of each specimen was done according to our clinical routine by specialized pathologists. Final histopathological diagnosis was evaluated according to current guidelines and was the reference standard in addition to post-operative PTH values.

The study was approved by the ethical review committee of canton Zurich (name: “Kantonale Ethikkommission Zürich”, number of application: KEK-ZH 2013–0556). No patient refused analysis of data; ethical approval included waiving of written informed consent because of retrospective, database driven approach and anonymization of patient records prior to analysis.

### Imaging

We scanned only patients without intravenous CT contrast or other excessive iodine intake recently (< 14 days). Two hours before scanning, patients received 20 MBq (±6%) of ^123^I sodium iodide orally. Ten minutes before image acquisition, each patient received 500 MBq (±6%) ^99m^Tc-tetrofosmin intravenously. SPECT imaging was acquired either on an Infinia Hawkeye 2 or Discovery NM 670 scanner (both GE Healthcare, Switzerland). Image acquisition was identical on both scanners. Simultaneous dual-energy windows were used for separated quantification of counts with 140 keV with total width of 15 keV (range 130–145 keV) for ^99m^Tc and 159 keV with total width of 15 keV (range 154–169 keV) for ^123^I. We used a circular orbit with dual detectors in 180° angle with 30 seconds per step and 3° per step (low-energy high-resolution collimators, matrix 128 x 128, pixel size 4.8 mm). Image reconstruction was performed on scanner console with scatter correction and OSEM (ordered subset expectation maximization) reconstruction with 2 iterations. A Hanning prefilter and a Butterworth postfilter were used in image postprocessing.

**Fig 1:**
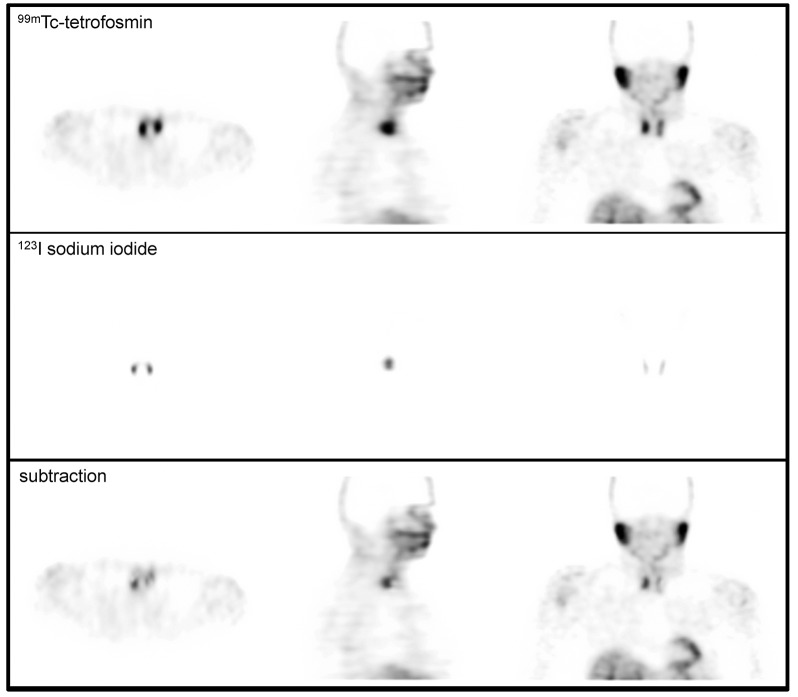
Example of dual-isotope ^99m^Tc-tetrofosmin and ^123^I sodium iodide single-photon-emission-computed-tomography (SPECT). ^**123**^I sodium iodide image (middle panel) was semi-manually normalized to ^**99m**^Tc-tetrofosmin image (upper panel) and afterwards subtracted from it (lower panel). Adenoma was detected at the right lower pole of the thyroid and patient received minimal invasive surgery (duration of surgery 90 minutes).

A board-certified nuclear medicine physician analyzed images blinded to results of operation. Image analysis was done with PMOD program (PMOD, Switzerland). ^123^I SPECT images were normalized to ^99m^Tc images by calculating the quotient between both images in a volume of interest (VOI) in the center of the thyroid by taking the mean value in this VOI. Each voxel of the ^123^I SPECT image was then divided by this factor. In a second step, each voxel of the normalized ^123^I SPECT image was subtracted from the corresponding ^99m^Tc SPECT image. Manual adjustment of normalization-factor was possible by the discretion of the rater to overcome over-subtraction [4]. A focal spot of residual activity on the subtraction image was defined as positive finding and allocated to an anatomical location (left and right upper pole, left and right lower pole and ectopic). An example is given in Fig 1.

### Statistics

Statistical analyses were performed using SPSS (version 21). Data were checked for normal distribution using Kolmogorov-Smirnov test (p > 0.05 used). For average comparison of normal distributed values, we used Student’s T-test, for non-normal distributed values Mann-Whitney-test, and for nominal values Chi-square test. All group data is described by mean and standard deviation.

## Results

Demographic and clinical characteristics of patients are summarized in Table 1. We included a total of 90 patients, 70 patients with p-HPT (mean age 56.6±12.5 years) and 20 patients with t-HPT (mean age 47.0±13.6 years). No patient experienced side effects from dual-isotope imaging. Patients with t-HPT were significantly younger and more equally distributed with regard to gender than patients with p-HPT (p = 0.004, and p = 0.011, respectively). T-HPT patients had higher levels of PTH (815.4±710.1 vs. 179.5±197.7 ng/l, p<0.001) and creatinine (492.1±450.0 vs. 76.6±18.6 μmol/l, p<0.001). Approximately 40% of all patients had concomitant thyroid disease; among these, 9 patients were on thyroid hormone replacement therapy for hypothyroidism and 29 patients had thyroid nodules.

**Table 1:** Demographic and clinical characteristics of patients.

	all	p-HPT	t-HPT	*p*
n	90	70	20	
***Demographics***				
Female	63 (70%)	54 (77%)	9 (45%)	**0.011**
Male	27 (30%)	16 (23%)	11 (55%)	
Age [years]	54.4±13.3	56.6±12.5	47.0±13.6	**0.004**
Concomitant thyroid disease	38 (42%)	29 (41%)	9 (45%)	0.13
***Pre-operative***	*(percent of patients with laboratory value)*		
PTH [ng/l]	320.8±456.8 (100%)	179.5±197.7 (100%)	815.4±740.1 (100%)	**<0.001**
Calcium [mmol/l]	2.8±0.3 (100%)	2.8±0.3 (99%)	2.5±0.3 (100%)	**<0.001**
Phosphate [mmol/l]	0.9±0.4 (93%)	0.8±0.3 (93%)	1.3±0.7 (95%)	**<0.001**
Creatinine μmol/l]	171.0±274.1 (98%)	76.6±18.6 (97%)	492.1±450.0 (100%)	**<0.001**
***Post-operative***	*(percent of patients with laboratory value)*			
PTH [ng/l]	45.3±71.3 (100%)	39.5±23.8 (100%)	66.6±145.6 (100%)	0.168
*with PTH ≤ 70ng/l*	*90%*	*94%*	*75%*	**0.023**
Calcium [mmol/l]	2.3±0.3 (99%)	2.4±0.1 (99%)	2.3±0.7 (100%)	0.100
*with calcium ≤ 2.54 mmol/l*	*92%*	*96%*	*80%*	**0.041**
Phosphate [mmol/l]	1.0±0.2 (74%)	1.0±0.2 (71%)	1.0±0.3 (85%)	0.871

PTH: parathyroid hormone, p-HPT: primary hyperparathyroidism, t-HPT: tertiary hyperparathyroidism

Normal range of values: PTH ≤ 70 ng/l, Calcium 2.09–2.54 mmol/l, Phosphate 0.87–1.45 mmol/l

SPECT was performed 4.8 months on average prior to surgery. After operation, calcium and PTH levels were available in 20 t-PTH (100%) and 69 p-HPT patients (99%), respectively. In p-HPT, 4 patients had elevated PTH levels post-operative, 2 patients had only slight elevation (77.5 and 83.1 ng/l) with regular calcium levels and further follow up of 6 months did not show a pronounced increase of PTH. The other 2 patients had stronger elevation (129.5 and 130.4 ng/l) but none of them elicited elevated calcium levels either. PTH levels resolved to normal in one patient during further follow-up of 6 months. In t-HPT, 5 patients elicited elevated PTH post-operative and 4 patients were hypercalcemic; all of these patients had partial resection and were treated conservatively during further follow-up.

In patients with p-HPT, SPECT suspected one pathological parathyroid lesion in 57 of 70 patients (81% defined as SPECT positive results). Pathology confirmed a pathological lesion in 53 of the 57 SPECT-positive lesions (= 93% positive predictive value, PPV); in 4 SPECT-positive lesions, regular parathyroid tissue was found at this site. In 50 of 57 patients (= 88%), SPECT-positive lesion was the only confirmed pathological parathyroid lesion. Patient-based sensitivity was 80%. In 13 of all 70 patients (19%), SPECT did not show suspicious focal uptake despite elevated PTH level at time-point of imaging. In a lesion-based approach, giving 5 possible locations for a lesion per patient (left and right upper pole, left and right lower pole and ectopic location: 5 locations x 70 patients = 350 possible locations), specificity was 99%. Sensitivity was 67% with stable positive predictive value of 93% on lesion-based analysis (Table 2).

**Table 2:** Contingency tables of patients with primary hyperparathyroidism.

*Patient based analysis*					*Lesion based analysis*				
		Pathology					Pathology		
		pos	Neg				pos	neg	
**SPECT**	pos	53	4	57	**SPECT**	pos	53	4	57
	neg	13	0	13		neg	26	267	293
		66	4	70			79	271	350

Pathology was counted as positive when a pathologic parathyroid was found at site of SPECT lesion

SPECT: single photon emission tomography, pos: positive, neg: negative.

Comparison of p-HPT patients with positive SPECT versus negative SPECT revealed significantly higher PTH levels (195.4±214.9 vs. 109.5±53.1 ng/l, p = 0.033) and higher weight of pathologic parathyroids (1.8±2.1 vs. 0.5±0.6 g, p = 0.007) in SPECT positive patients (Table 3). In these patients, underlying pathology was more often an adenoma than a hyperplastic parathyroid compared to patients with negative SPECT (p = 0.021). Total duration of parathyroid surgery was 40 minutes shorter on average in SPECT-positive than in SPECT-negative patients (89±46 vs. 129±4 minutes, p = 0.006). Eighty-six percent of these patients received minimal invasive parathyroidectomy, and only one of them had elevated PTH during follow-up. In SPECT-negative patients minimal invasive surgery was performed in 50% (p = 0.021).

**Table 3:** Differences in demographics, laboratory values, gland weight and duration of surgery in SPECT- positive and - negative p-HPT subjects in patient based analysis.

	SPECT positive	SPECT negative	*p*
n	57	13	
Age [years]	55.8±12.4	59.7±13.2	0.317
Concomitant thyroid disease	37%	62%	0.625
***Pre-operative***	*(percent of patients with laboratory value)*		
PTH [ng/l]	195.4±214.9 (100%)	109.5±53.1 (100%)	**0.033**
Calcium [mmol/l]	2.8±0.3 (100%)	2.8±0.3 (92%)	0.101
Phosphate [mmol/l]	0.8±0.3 (93%)	0.8±0.1 (92%)	**0.045**
***Post-operative***	*(percent of patients with measurement)*		
Weight of pathologic parathyroid [g]	1.8±2.1 (91%)	0.5±0.6 (77%)	**0.007**
Histopathologic diagnosis:			
Adenoma	60%	15%	**0.021**
Hyperplasia	33%	77%	
***Surgery***	*(percent of patients with measurement)*		
Duration of surgery [min]	89±46 (88%)	129±41 (77%)	**0.006**

PTH: parathyroid hormone

Normal range of values: PTH ≤ 70 ng/l, Calcium 2.09–2.54 mmol/l, Phosphate 0.87–1.45 mmol/l

In 20 patients with t-HPT, SPECT was positive in 12 patients (60%). Ninety percent of patients with t-HPT had more than one pathologic parathyroid in histopathology and all of these patients had bilateral lesions. Sensitivity of SPECT was 61%, specificity was 50%, and positive predictive value was 92% in patient-based analysis. In lesion-based analysis, SPECT had sensitivity of 25% for detecting all lesions, specificity was 98% and positive predictive value was 93% (Table 4).

**Table 4:** Contingency tables of patiens with tertiary hyperparathyroidism.

*Patient based analysis*					*Lesion based analysis*				
		Pathology					Pathology		
		pos	neg				pos	neg	
**SPECT**	pos	11	1	12	**SPECT**	pos	14	1	15
	neg	7	1	8		neg	43	42	85
		18	2	20			57	43	100

Pathology was counted as positive when a pathologic parathyroid was found at site of SPECT lesion

SPECT: single photon emission tomography, pos: positive, neg: negative.

## Discussion

Our study on 90 patients with hyperparathyroidism demonstrates high diagnostic value of dual-isotope ^99m^Tc-tetrofosmin and ^123^I sodium iodide SPECT in primary hyperparathyroidism and provides evidence for substantially shorter duration of surgery in SPECT-positive patients. In tertiary hyperparathyroidism, however, diagnostic performance of dual-isotope SPECT was considerably lower in our cohort.

Up to date, multiple different scintigraphic and SPECT-based methodologies for detecting pathological parathyroids have been described, including dual-phase, dual-isotope, planar and CT combined imaging approaches, with wide range of sensitivity and specificity for each method [4, 6, 11–14]. Different approaches in calculation of sensitivity and specificity were used with different references as gold standard. Especially, selection of gold standard for true negatives (meaning physiological, “healthy” parathyroid glands) is challenging as the desired histopathological proof of all parathyroids in each patient is not achievable. Nevertheless, taking different standards for calculation can result in difficulties in comparison of results [8, 11]. Therefore, we calculated a patient-based analysis and a lesion-based approach including all possible locations. Correct identification of all pathological parathyroids by surgery in each patient was ascertained by follow-up PTH and calcium testing. In p-HPT, calculated sensitivity of the dual-isotope ^99m^Tc-tetrofosmin and ^123^I sodium iodide SPECT was 80% and in the upper range of previously published data with a very high PPV of 93% [4, 15]. Ninety-four percent of patients had normal values of PTH after surgery, pointing to a high rate of cure and good feasibility of used gold standard. Four patients had elevated levels, but 2 patients had only mild elevation without any further increase in the next 6 months. These 2 patients might therefore be stated as cured, too. The other 2 patients had stable PTH levels >100 ng/l during the next 6 months. As all patients had stable normal levels of calcium, no further surgery was indicated and no follow-up image was performed. Discordance of elevated PTH and normal calcium levels might represent pathophysiological adaptation.

Only 4 patients had a false positive scan with regular parathyroid tissue at site indicated by SPECT. Due to the very low pre-test probability of patients without any parathyroid pathology (as all patients had elevated PTH and clinical diagnosis of p-HPT), these 4 scans resulted in low specificity in patient based analysis as no patient was without pathological parathyroid gland and counted as true negative. In the lesion-based approach with more realistic pre-test probability, specificity was excellent with 99% taking 5 possible sites and still 99% if ectopic position was excluded. This is higher than previous reports for different imaging approaches [8, 14].

In our analysis of influencing factors of parathyroid detection in p-HPT, patients with a positive ^99m^Tc-tetrofosmin and ^123^I sodium iodide SPECT scan had higher pre-operative levels of PTH and higher weight of pathological parathyroids than patients with negative SPECT. This relationship is reported for dual-isotope ^99m^Tc-sestamibi and ^123^I sodium iodide imaging as well as for ^99m^Tc-sestamibi dual-phase scanning [3, 8, 16, 17]. As higher levels of PTH points to a higher biological activity of the lesion, a stronger uptake of the mitochondrial tracers tetrofosmin and sestamibi can be assumed from pathophysiological considerations. We observed also a better detection of parathyroid adenomas than parathyroid hyperplasia. Lower sestamibi uptake was reported for parathyroid hyperplasia compared to adenomas which might be due to different biochemistry of these entities [17, 18]. As tetrofosmin, used in our study, has similar uptake into parathyroid lesions as sestamibi, mentioned results obtained for sestamibi in hyperplasia might be transferable to tetrofosmin [9]. This would be in line with our results of significant higher percentage of hyperplastic parathyroids in SPECT negative patients.

In our p-HPT cohort, a relatively high proportion of 41% of patients had thyroid disease that is similar to the rate reported by Hassler and colleagues who did find a marked decrease in sensitivity to 62% compared to 85% in patients without thyroid disease [11]. Concomitant thyroid nodules might elicit increased or decreased iodine uptake, thereby perturb subtraction image. Supra-physiological thyroid hormone intake and hypothyreoidism can lead to decreased iodine uptake; however, we did not observe hampered uptake in critical re-analysis of images. Although patients with SPECT-negative lesions tend to have more often concomitant thyroid disease, differences were not statistical significant in our analysis. In a more detailed study on concomitant thyroid diseases by Rink and colleagues, only size of the thyroid influenced sensitivity and specificity but not morphological or functional alterations; this might explain differences [19]; however, we did not calculate total thyroid volume in our analysis.

Our study indicated that pre-operative dual-isotope ^99m^Tc-tetrofosmin and ^123^I sodium iodide SPECT imaging can substantially save time of operation in patients with positive SPECT, on average 40 minutes. Cost-calculations for operation time assume € 17–18 per minute in Switzerland which is supposed to be in similar range in other European countries [20]. As SPECT was positive in 81% of patients with p-HPT and duration of surgery was 40 minutes shorter in these patients, this resulted in 2280 saved minutes and € 41’000.- in our cohort of 70 patients. Shorter operation time is achieved by minimal invasive parathyroidectomy, done in 86% of SPECT-positive patients in our cohort, which is favored by endocrine surgeons not only due to faster intervention but lower risk of complications [4, 21]. Guiding principle for this approach is the need of a high PPV of indicated lesion by SPECT which was 93% in our analysis. In our study, endocrine surgeons could decide surgical approach in each patient. Therefore, the shorter duration and implementation of minimal invasive parathyroidectomy in 86% of SPECT positive patients reflects high acceptance of pre-operative parathyroid imaging for minimal invasive surgery planning. High rate of post-operative cure in this group affirms this approach which was not inferior to the reported 95% cure in bilateral cervical neck exploration without pre-operative imaging in p-HPT [22]. Hence, our results confirm feasibility and cost-efficiency of pre-operative imaging with dual-isotope ^99m^Tc-tetrofosmin and ^123^I sodium iodide SPECT in patients with p-PHT as reported for other tracers [4, 23].

In patients with t-HPT, dual-isotope ^99m^Tc-tetrofosmin and ^123^I sodium iodide SPECT had lower sensitivity of 61% in patient based-analysis. In our cohort, 90% of patients had more than one lesion and all of these patients had lesions on both sides, therefore, minimal-invasive surgery is only feasible in the very minority of patients. Nevertheless, pre-operative knowledge of all pathologic lesions might increase surgical accuracy. Published data on post-operative cure in t-HPT state higher rate of recurrence similar to our analysis with a lower rate of approximately 80% compared to patients with p-HPT [4]. Higher rate of post-operative elevated PTH might be the consequence of intentional subtotal parathyroidectomy in patients with renal failure [24], and the subset of four patients with hypercalcemia in our analysis underwent only partial resection of parathyroid tissue, too. In current guidelines, the value of pre-operative imaging is still considered unclear and undetermined; bilateral cervical exploration still remains the standard surgical approach [3, 15]. Sensitivity for detection all lesions in patients with t-HPT in our cohort was rather disappointing with only 25% which may be due to the fact that patients with false-negative scans more often had 3 or 4 pathological lesions, with glandular hyperplasia as most common histopathological finding. Other authors reported superior diagnostic performance with nearly 80% sensitivity, especially for dual-isotope ^99m^Tc-sestamibi and ^123^I sodium iodide imaging [25, 26]. Noticeable in our analysis, only few images were judged as showing multiple lesions, reflecting a possible pitfall in subtraction imaging when over-subtraction occurs and lesions with only subtle increased uptake are deleted [4]. Newer positron emission tomography (PET) tracers like ^11^C-methionine and ^18^F-choline have been reported to have higher uptake in glandular hyperplasia and in SPECT-negative lesions. Therefore, these tracers might allow better diagnostic accuracy in t-HPT; however, experience and availability is limited [13, 27, 28].

We did not use co-registration with CT which might be one limitation of our study. However, use of CT can improve anatomical localization but changes of diagnosis were rare in a head-to-head comparison to SPECT imaging only [29, 30]. Another limitation of our study is the relative short duration of the laboratory follow-up of only 3 months. In a large analysis of patients undergoing surgery for p-HPT, however, only 3 of 656 patients developed elevated PTH levels after 6 months despite regular levels 10 days after operation [1].

In conclusion, dual-isotope SPECT with ^99m^Tc-tetrofosmin and ^123^I sodium iodide has a high diagnostic value in patients with primary hyperparathyroidism and allows for saving of operation time and costs. Pre-operative value of dual isotope SPECT in tertiary hyperparathyroidism remains uncertain but newer PET tracers might overcome shortcomings in diagnostic sensitivity.

## Supporting Information

S1 STARD Checklist (DOC)
